# Dietary Patterns Are Associated with Risk of Prostate Cancer in a Population-Based Case-Control Study in Montreal, Canada

**DOI:** 10.3390/nu12071907

**Published:** 2020-06-27

**Authors:** Karine Trudeau, Marie-Claude Rousseau, Christine Barul, Ilona Csizmadi, Marie-Élise Parent

**Affiliations:** 1 Epidemiology and Biostatistics Unit, Centre Armand-Frappier Santé Biotechnologie, Institut National de la Recherche Scientifique, University of Quebec, Laval, QC H7V 1B7, Canada; karine.trudeau@iaf.inrs.ca (K.T.); marie-claude.rousseau@inrs.ca (M.-C.R.); christine.barul@iaf.inrs.ca (C.B.); 2 School of Public Health, Department of Social and Preventive Medicine, University of Montreal, Montreal, QC H3N 1X9, Canada; 3 University of Montreal Hospital Research Centre, Montreal, QC H2X 0A9, Canada; 4 Department of Surgery, Cedars-Sinai Medical Center, Los Angeles, CA 90048, USA; ilona.csizmadi@csmc.edu; 5 Department of Community Health Sciences, Cumming School of Medicine, Calgary, AB T2N 4N1, Canada

**Keywords:** prostate cancer, case-control study, dietary patterns, Healthy Eating pattern, Western Salty and Alcohol pattern, Western Sweet and Beverages pattern, principal component analysis

## Abstract

This study describes the association between dietary patterns and prostate cancer (PCa) risk in a population-based case-control study conducted in Montreal, Canada (2005–2012). Cases (*n* = 1919) were histologically confirmed, aged ≤75 years. Concomitantly, controls (*n* = 1991) were randomly selected from the electoral list and frequency-matched to cases by age (±5 years). During face-to-face interviews, a 63-item food frequency questionnaire focusing on the two years before diagnosis/interview was administered. Three dietary patterns were identified from principal component analysis. Unconditional logistic regression estimated the association between dietary patterns and PCa, adjusting for age, ethnicity, education, family history, and timing of last PCa screening. When comparing scores in the highest vs. lowest quartiles, the Healthy Eating pattern was associated with a decreased risk of overall PCa (Odds ratio (OR) = 0.76, 95% confidence interval (CI) = 0.61, 0.93); this association was stronger for high-grade cancers (OR = 0.66, 95% CI = 0.48, 0.89). By contrast, the Western Sweet and Beverages pattern was associated with an elevated risk of overall PCa (OR = 1.35, 95% CI = 1.10, 1.66). The Western Salty and Alcohol pattern was not associated with PCa risk. These findings suggest that some dietary patterns influence PCa development.

## 1. Introduction

A role for diet in the development of prostate cancer (PCa) has long been suspected. It has been proposed as a potential explanation for the persistent geographic distribution of the incidence of this cancer and by studies of migrant populations [1,2,3,4,5]. However, the evidence on this issue remains weak and inconsistent [5]. Several methodological issues may have hampered the ability to identify associations with dietary factors, including limited exposure assessment and study size, as well as the lack of consideration of tumor aggressiveness. Moreover, many case-control and prospective studies were likely subject to detection bias, as latent, undiagnosed cancers may have been included among non-cases. PCa screening, which may be associated with lifestyle and diet [6], has been largely overlooked in previous studies.

The most common approach used in epidemiological investigations of diet in PCa has involved the study of a single nutrient or food group at a time [5,7,8]. An analytical approach based on dietary patterns has recently been proposed, which may be more relevant from an etiological point of view [9,10]. Dietary patterns take into account that foods and nutrients are consumed as meals and snacks rather than as single items and nutrients in isolation [9]. A number of studies have been published on the association between dietary patterns derived from data-driven or a posteriori methods [11] and risk of PCa [12,13,14,15,16,17,18,19,20,21,22,23,24,25,26]. Some have found that dietary patterns characterized by consumption of meat or a Western pattern were associated with an increased risk of PCa [12,18,20,23,25], while others observed that a Healthy pattern or a Mediterranean diet reduced its risk [14,15,18,22]. In a handful of studies, PCa risk was analyzed according to the aggressiveness of the tumors, and results vary widely. Western and Refined Carbohydrate patterns have been associated with aggressive and low-grade PCa, respectively [12,17], while a Mediterranean diet reduced the risk of aggressive cancers [15]. By contrast, no association has emerged with dietary patterns and PCa aggressiveness in another investigation [19].

Montreal, Canada harbors a distinct cultural heritage with a majority of residents of French descent. This is reflected in the diet, with traditional dishes borrowed from their founders, mixed with multicultural influences. It is thus of interest to study dietary patterns among Montrealers in relation to PCa, which is the most common cancer among men in this population, after non-melanoma skin cancer [27].

We hypothesized that a dietary pattern reflecting the more traditional cuisine, which is rich in meat, would be associated with a higher risk of PCa, while a dietary pattern rich in fruits and vegetables would be associated with a lower risk of the cancer. The objective of the current study was to evaluate the role of dietary patterns on the risk of PCa in the context of a large population-based case-control study conducted in Montreal, Canada.

## 2. Materials and Methods

### 2.1. Study Population

The current analyses are based on the Prostate Cancer and Environment Study (PROtEuS). This investigation, described previously [28], aims to identify environmental, occupational, lifestyle, and genetic factors in the development of PCa. Briefly, eligibility criteria for cases and controls included Canadian citizenship, registration on the provincial electoral list, residence in the Montreal metropolitan area, and being ≤75 years of age at diagnosis or time of interview (index date). Cases were diagnosed with primary, histologically confirmed PCa between September 2005 and December 2009, ascertained through pathology departments across 7 of 9 French-language hospitals in Montreal. Comparisons with the provincial tumor registry showed that ascertained cases represented 80% of all cases diagnosed in the area during the accrual period. Concurrently, population controls were randomly selected from Quebec’s permanent electoral list of French electors and frequency-matched to cases by age (±5 years). Eligible controls had no history of PCa at the time of recruitment. In all, 1932 cases and 3026 controls were ascertained. Among eligible subjects, responses rates were 79% for cases and 56% for controls. Reasons for non-participation were refusal (94% and 86%), unable to contact (3% and 11%), death (2% and 1%), or too sick to participate with no proxy available (1% of controls), and language barrier (1% and 1%), among cases and controls, respectively. For less than 4% of subjects, the interview was conducted with a proxy respondent, usually the spouse.

PROtEuS was approved by the Comité d’éthique en recherche avec les êtres humains of the Institut national de la recherche scientifique (CÉR-02-036, from October 8, 2002 till present), as well as by the ethics boards of all participating hospitals. All subjects provided written informed consent.

### 2.2. Data Collection

In-person interviews, conducted between 2005 and 2012, elicited information on sociodemographic, environmental, medical, and lifestyle factors including smoking history, alcohol use, coffee and tea consumption, and diet. Dietary information was obtained with a 63-item food frequency questionnaire (FFQ) which is based on a validated instrument used by the Canadian Cancer Registries Epidemiology Research Group, with slight modifications to reflect the specificity of the study population [29]. Diet was assessed for the period two years prior to the index date and reflected food consumed at home, work, and in restaurants. Dietary data were missing for 13 cases (0.7%) and 3 controls (0.01%) interviewed, and 42 controls became cases of PCa during the ascertainment period, resulting in 1919 cases and 1991 controls for analyses. The consumption of food items was queried in terms of the frequency of use per day, week or month, based on commonly used portion sizes. Participants were also asked about seasonal variations in the consumption of various fresh fruits. Additional questions probed for further details, such as the consumption of fat from meat, skin of poultry and cooking methods. Use of coffee, black tea, green tea, beer, wine, and spirits two years before the index date was also determined. A separate set of questions inquired whether subjects had modified their intake of fruits, vegetables, red meats, other meats, cereal products, dairy products, fat (butter, oil), sweets and deserts, alcohol, coffee, and tea in the period between 20 years and 2 years before the index date.

Information on the frequency of PCa screening by prostate-specific antigen (PSA) and digital rectal examination (DRE) in the previous 5 years was recorded. Gleason scores, used to indicate tumor aggressiveness, were extracted from diagnostic biopsy pathology reports. A Gleason score of 6 or lower, or a score of 7 with a primary score of 3, corresponding to a low-grade tumor, indicated a non-aggressive cancer; a Gleason score of 7 with a primary score of 4, or 8 or higher, reflecting a high-grade tumor, was indicative of an aggressive cancer [30].

### 2.3. Statistical Analysis

Principal component analysis (PCA) was used to identify dietary patterns using the control series, as previously described in Trudeau et al. [31], albeit based on a slightly different study population. The analysis was performed on the correlation matrix comprised of the 72 following variables: the 63 items from the FFQ, the consumption of coffee, black tea, green tea, beer, wine, and spirits, and three complementary questions relating to the consumption of fat from meat, skin of poultry, and cooking methods. Since the distribution of the variables was skewed, log transformation was compared to untransformed data and judged to be preferable. A component was retained when it met all of the following criteria: eigenvalue > 2.0, identification of a breaking point in the scree plot, a sufficient proportion of variance was explained, and factor interpretability [32].

Variables with an absolute factor loading ≥ 0.2 were considered to load on a component [32]. Dietary patterns were labelled according to the main food items loaded on a retained component. As an additional assessment of the robustness of the dietary patterns identified, we re-ran the analysis by randomly placing subjects into one of two equal-sized groups, or split-samples, which led to comparable results. We assessed the effect of changes in food consumption over the last 20 years on our estimates, using the set of questions specifically designed to capture dietary changes. Based on dietary survey data from Quebec residents [33], we converted the weekly frequency of use of all items according to the reported corresponding changes (increase, decrease, same) in food consumption over the last 20 years. We then ran an analysis with a model taking into account the changes in foods habits over the previous 20 years; the data did not provide a different solution. The factor score for each dietary pattern was computed by determining the optimal regression weights, multiplying subjects’ answers to the questionnaire items by these weights and summing the products. Each dietary pattern factor score was categorized into quartiles based on the distribution among controls.

Unconditional logistic regression was used to estimate odds ratios (ORs) and 95% confidence intervals (CIs) for the association between the score for each dietary pattern, and PCa risk. Unconditional polytomous logistic regression was used to estimate risks by cancer aggressiveness. Dose-response relationships were tested by modeling each category as a continuous variable.

**Figure 1:**
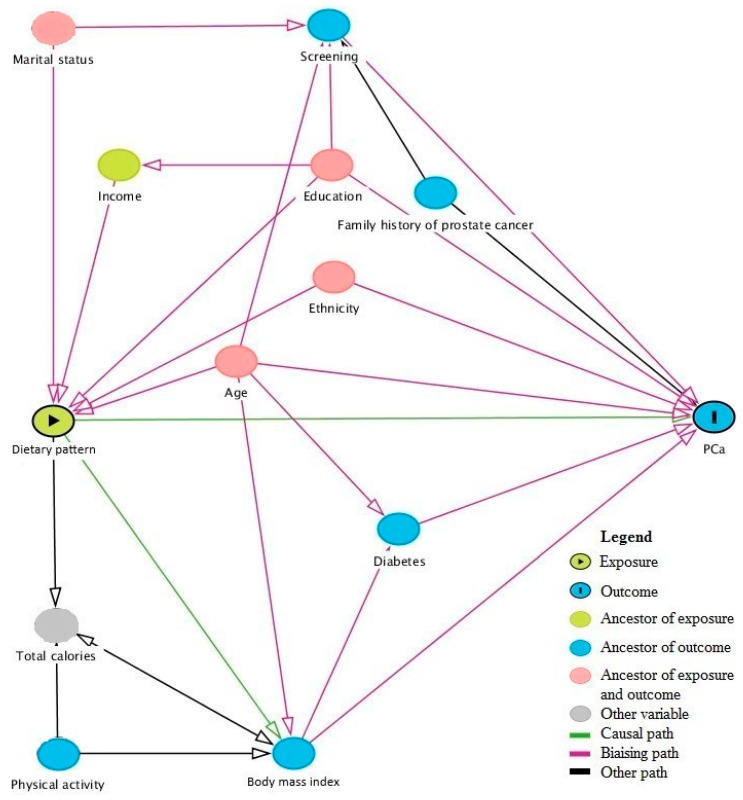
Directed acyclic graph for the association between dietary patterns and prostate cancer risk.

In order to identify potential confounders, we drew a directed acyclic graph (DAG) for the association between dietary patterns and PCa (Figure 1) representing the total effects [34]. Variables considered in the DAG included age at diagnosis for cases or age at interview for controls (continuous), ethnicity (Asian, Sub-Saharan, European, Greater Middle Eastern, Latino), education (elementary or less, high school, college, university, other), first-degree family history of PCa (yes, no, do not know), timing of the last prostate screening by PSA and/or DRE (≤2 years before index date, >2 years before index date, never screened, do not know), marital status (married or common law, separated or divorced, single, widower, member of a religious order, do not know), family income (<20,000$CAD, 20,000–29,999$CAD, 30,000–49,999$CAD, 50,000–79,999$CAD, >80,000$CAD), diabetes (yes, no, do not know), body mass index (BMI, continuous), total caloric intake (kcal/day, continuous), and physical activity (not very active, moderately active, and very active). Total effects (shown in pink), which is more commonly presented, closes all biasing paths and leaves all causal paths open. A minimal model included age, education, ethnicity, and marital status. We chose to present here results based on a more complete and etiologically relevant model, which includes age, education, ethnicity, first-degree family history of PCa and timing of last PCa screening test. We considered creating a causal link for BMI, but this would not have altered findings.

Finally, we conducted a sensitivity analysis excluding controls who had not been screened for PCa in the previous 2 years and who were more likely to have a latent, undiagnosed PCa.

All analyses were performed using SAS (version 9.4; SAS Institute, Cary, NC, USA.) A *p*-value of less than 0.05 indicated statistical significance.

## 3. Results

### 3.1. Study Population

Table 1 shows selected characteristics of the 1919 cases and 1991 controls. The mean ages of cases and controls were 64 and 65 years, respectively, owing to the slightly longer time required to recruit controls into the study. Most subjects were of European ancestry. Subjects were overweight, on average, two years prior to the index date, based on the World Health Organization classification for obesity [35]. Compared to controls, cases were less educated and more often of European or African ancestry, while less often of Asian or Greater Middle Eastern ancestry, and had more often a first-degree family history of PCa. Nearly all cases and 76% of controls had undergone prostate screening by PSA testing and/or DRE in the two years prior to the index date. Cases and controls did not differ in terms of family income, cigarette smoking, physical activity, use of vitamin or mineral supplements, and total caloric intake two years before the index date.

Based on data derived from the Canadian Census for 2008, the percentages of subjects living in areas with a greater proportion of recent immigrants were 5% and 6%, for participants and non-participants, respectively. Corresponding values were 7% and 7% for higher unemployment rate, 19% and 20% of adults without a high school diploma, and 22% and 25% in the lowest quintile of household income, suggesting a very slight trend towards a higher socio-economic status among participants.

### 3.2. Identification of Dietary Patterns

The determinant of the correlation matrix, derived from PCA, was 9.19 × 10 ^−6^, indicating that the correlation matrix was neither a singular matrix nor an identity matrix. Furthermore, Bartlett’s test of sphericity was statistically significant (*p* < 0.0001), against the hypothesis that the correlation matrix is an identity matrix [32]. The global MSA was 0.84, which is meritorious according to Kaiser [36]. Four variables (margarine on bread, potatoes or vegetables; butter on bread, potatoes or vegetables; consumption of skin on poultry; and black tea) had a MSA < 0.5 and were not retained for subsequent analyses, leaving 68 dietary variables. After an orthogonal rotation, a three-component solution emerged; results are shown in Table 2.

The eigenvalues for those three components were 5.86, 4.92, and 2.48, and the proportion of the variance explained by each was 8.6%, 7.2%, and 3.7%, respectively, for a total of 19.5% of the variance in the diet accounted for in the study population. The three retained components were identified as distinct dietary patterns, labelled as Healthy Eating, Western Salty and Alcohol, and Western Sweets and Beverages, respectively. The Healthy Eating pattern was characterized by a high consumption of fruits, vegetables, tofu, soybeans, fish, brown bread, nuts or peanut butter, yogurt, and no consumption of white bread. The Western Salty and Alcohol pattern included high loadings for beef, pork, chicken, veal, lamb, hot-dogs or sausages, cold cuts, bacon, breakfast sausage, barbecue cooking, white bread, fat of beef or pork, meat slightly blackened, beer, wine, spirits, and no consumption of brown bread, tofu or soybeans. The third pattern, Western Sweets and Beverages, reflected high loadings of pasta with tomato sauce, pasta with cheese, pizza, cookies, muffins, donuts, cakes, pastries, pies, oatmeal or cream of wheat, breakfast cereal, chips, corn chips, popcorn, tortillas, chocolate, ice cream, tomato or vegetable juice, glass of milk or milk in cereal, dark carbonated soft drinks, and other carbonated soft drinks.

### 3.3. Association between Dietary Patterns and PCa Risk

Table 3 present results for the association between dietary pattern scores, in quartiles, and the risk of PCa overall and by tumor grade based on total effects, as identified with the DAG. Analyses were also conducted using the minimal model and results were virtually the same (Supplementary Table S1).

In analyses comparing scores in the highest versus the lowest quartiles, the Healthy Eating pattern was consistently associated with ORs below unity, e.g., overall PCa, OR = 0.76 (95% CI = 0.61, 0.93); low-grade PCa, OR = 0.90 (95% CI = 0.71, 1.13); high-grade PCa, OR = 0.66 (95% CI = 0.48, 0.89), suggesting a protective effect of this dietary pattern, particularly pronounced for aggressive cancers. Moreover, the closer subjects adhered to the Healthy Eating pattern, the stronger the inverse association was for overall (*p*-trend = 0.004) and high-grade (*p*-trend = 0.008) PCa. Conversely, ORs for the Western Sweet and Beverages pattern were higher in the fourth quartiles as compared to the first, suggesting an increase in PCa risk associated with this pattern: overall PCa, OR = 1.35 (95% CI = 1.10, 1.66); low-grade PCa, OR = 1.14 (95% CI 0.91, 1.44); high-grade PCa, OR = 1.32 (95% CI = 0.98, 1.80). There was also evidence that increasing adherence to this pattern was associated with increasing risk, especially for overall (*p*-trend = 0.002) and high-grade (*p*-trend = 0.02) cancers. Lastly, the Western Salty and Alcohol pattern was not associated with PCa risk.

In sensitivity analyses excluding controls not screened for PCa in the previous two years, thereby limiting the likelihood of latent PCa in the control series, results were similar to those in the main analyses (Supplementary Table S2).

## 4. Discussion

In this study, we observed an inverse association between adherence to the Healthy Eating pattern and overall, as well as high-grade PCa, with evidence of an exposure-response pattern. In contrast, adherence to the Western Sweet and Beverages pattern was associated with a higher PCa risk overall and for high-grade tumors. Increasing adherence to this pattern increased risk. No clear association was found in relation to the Western Salty and Alcohol pattern.

### 4.1. Previous Studies

#### 4.1.1. Cohort Studies

To date, five prospective cohort studies conducted in the US [13,24,26], Japan [23], and Australia [19] have investigated the role of dietary patterns in PCa risk. Three were suggestive of no association [19,24,26]. The first negative study, the Health Professionals Follow-up study (3002 cases), assessed adherence to Prudent and Western patterns [26]. The second, the Melbourne Collaborative Cohort study (1018 cases), reported no association with four dietary patterns, e.g., Mediterranean, Vegetable, Meat and Potatoes, and Fruit and Salad [19]. In the third cohort study, the National Health and Nutrition Examination Survey Epidemiological Follow-up Study (136 cases), there were no associations with a Vegetable and Fruit and a Red Meat and Starch pattern, although men in the highest tertile of adherence to the Southern dietary pattern had a slightly lower risk of PCa [24].

In contrast, two other cohort studies document associations between dietary patterns and PCa. The largest, the National Institutes of Health (NIH)-AARP Diet and Health Study [13], included 23,453 cases. Men in the highest quintile of the Healthy Eating index-2005 and of the alternate eating index-2010 had a reduced risk of PCa, but no association emerged with the Mediterranean diet score. In the second study, conducted in Japan (1156 cases), men adhering to a Western pattern had an elevated PCa risk, those adhering to a Prudent pattern had a lower risk and there was no association with a Traditional pattern.

#### 4.1.2. Case-Control Studies

In all, nine case-control studies have investigated the role of dietary patterns in PCa. The largest (1294 cases, 1451 controls) identified five nutrient-based dietary patterns [21]. Elevated risks were associated with Animal Products, Starch-rich and Unsaturated Fatty Acids patterns while no association was found with Vitamins and Fiber and Vegetable Unsaturated Fatty Acids patterns. The second largest case-control study [15], the MCC-Spain study (733 cases and 1229 controls), suggested a protective effect of the Mediterranean diet among men with Gleason scores > 6. The Western and Prudent patterns were not associated with PCa risk [15]. Of note, in that study dietary patterns were derived from dietary intakes reported by women in another Spanish study [37]. The third largest study (546 cases and 447 controls) was conducted in Western Australia [12]. The Western pattern was associated with an increased risk of PCa while the Vegetable and Health-Conscious patterns were not associated with risk.

Six other smaller case-control studies, including less than 500 PCa cases, have documented positive associations with various dietary patterns, including Refined Carbohydrates and Sweet Baked Foods [17], traditional Uruguayan and Western [16], Processed Diet [25], Carbohydrate, and Traditional [20]. Interestingly, none of these studies observed inverse associations with the dietary patterns identified.

In summary, results from three studies are consistent with a protective effect of “healthier” diets against PCa [13,15,23] while six studies suggest a harmful effect of diets rich in meat, such as the Western diet, on overall or aggressive PCa [12,16,18,20,21,23]. Our findings of a protective association with the Healthy Eating pattern, and of a positive association with the Western Sweet and Beverages pattern, with respect to overall and high-grade cancers, thus re-enforce the general tendencies already observed.

### 4.2. Methodological Considerations

Our study, including 1919 cases and 1991 controls, is the largest population-based case-control study to date to assess the role of dietary patterns among predominantly Caucasian men in PCa risk. It was conducted in Montreal, which harbors a dietary culture with a strong French influence. The dietary patterns identified here, as those in all other studies, reflect local cultural heritages, so perfect alignment of patterns across various study populations is not expected.

In studies of diet and chronic diseases, prospective designs are usually preferred, provided that that follow-up is long enough to allow for a sufficient number of cases to accrue. This may pose a challenge to the study of PCa, which generally develops at an advanced age, requiring prolonged follow-up. Unlike for case-control studies, which are subject to reporting bias, the prospective nature of previous cohort studies conducted on this issue alleviates concerns for recall bias based on disease status. However, in cohort studies investigating dietary patterns, assessments were typically conducted at study baseline among men of various ages, sometimes ranging as far apart as from ages in the 20s to the 80s, without accounting for changes in food consumption over time. The timing of the assessment in cohort studies may thus have reflected remote or recent intakes, depending on the study, whereas in all case-control studies, including ours, reports focused on recent diet, which may be more relevant to cancer progression. We were also able to capture changes in dietary intake of several key food groups within the 20 years preceding the index date. In analyses restricting subjects to those reporting no major changes in intakes over this period, results were not altered (data not shown).

There necessarily was some degree of misclassification of dietary exposures in our study. As in previous investigations, we used a validated FFQ, recognized as a standard method of dietary assessment for determining usual food intake [38]. The questionnaire focused on the two years before diagnosis/interview to reduce the likelihood that cancer patients would report diets reflecting dietary changes post-diagnosis.

The majority of previous studies [12,19,21,24,26], like ours, used PCA to derive dietary patterns. Others used exploratory factor analysis [23] or pre-defined patterns such as eating indices [13]. Our sample size was sufficiently large to use PCA with 72 dietary items which presented several advantages. PCA alleviates the problems of collinearity between the foods consumed concurrently [39]. Moreover, it enables the use of continuous variables, which are estimated with FFQs, without having to create categorical variables, thereby reducing exposure misclassification.

In our study, the pattern explaining the highest percentage of variance of the food intake was Healthy Eating (8.6%), followed by Western Salty and Alcohol (7.2%) and Western Sweet and Beverages (3.7%), resulting in 19.5% of the total variance explained. This is similar to several other investigations where patterns explained around 11–38% of the total variance [12,17,23,25], albeit less than in the study on nutrient-based patterns, explaining 78% of variance in nutrient intake [21]. Of interest, the food items constituting the Health Eating pattern are aligned with the recommendations from Canada’s Food Guide [40].

Participation rates were imperfect, albeit relatively good as compared to several previous investigations [41]. Our comparison of participants and non-participants, separately for cases and controls, to census-derived variables indicated minimal differences between groups, consistent with the absence of a major selection bias. Selection bias based on food consumption is implausible as there was no mention to potential participants that the study included a dietary component.

Information on several covariates was available in the study, enabling their consideration when building our analytical models. Nevertheless, the potential for residual confounding or confounding by an unmeasured factor remains possible. For instance, physical activity may influence PCa risk indirectly through BMI or directly via various changes in other metabolic patterns not considered here [42]. However, in a recent review of the association between physical activity and metabolic syndrome, physical activity was more often associated with a reduction in obesity (waist circumference) than with a resolution of other components of the syndrome [43]. Furthermore, the American Institute of Cancer Research in their most recent update of the evidence for physical activity and prostate cancer risk has classified the evidence in the category of “limited – no conclusion” [5].

While several statistical tests were conducted as part of this study, we did not implement corrections for multiple testing. The dietary patterns identified were derived using PCA and were not pre-defined based on a priori information, resulting in novel patterns specific to this study population and for which associations with PCa had never been evaluated. Therefore, given the hypothesis-generating nature of the study, it was judged preferable to accept the possibility that some of the associations observed might have occurred by chance, based on the premise that findings should be confirmed in future studies [44,45].

Two large cohort studies suggest a protective effect of a “healthy or prudent” diet in PCa risk [13,23]. This is the first case-control study to report a similar finding, in further support of the WHO’s recommendations of eating fruits and vegetables to reduce the risk of disease [46]. Since they consider that foods are eaten together, and not alone, food patterns may be more easily amenable to health promotion interventions and chronic disease prevention.

Our findings document stronger associations, either protective (Healthy Eating) or harmful (Western Sweet and Beverages) with high-grade cancers, as compared to low-grade ones. Other studies also suggest associations that are specific to aggressive or advanced cancers, such as a lower risk with a Mediterranean pattern [15] and a higher risk with a Western pattern [16]. Ambrosini et al. found a higher risk among men with a Western pattern; results were attenuated for non-aggressive cancers [12]. By contrast, Jackson et al. observed a higher risk with a Refined Carbohydrate pattern and results were more pronounced for low-grade PCa [17]. There is evidence that less aggressive and more aggressive cancers may have different sets of risk factors and etiology [5,47]. Indeed, low-grade and high-grade cancer foci progress largely in parallel, diverging early from a common progenitor [48].

At the time our study was conducted, there was a very high uptake of PCa screening in the study base, despite the absence of a screening program. Screening was often integrated in routine yearly exams in this population with a free, universal access to healthcare. This distinguishes this study from many others, as detection issues can bias associations between exposures, including diet, and PCa [6]. For instance, health-conscious individuals may tend to have both a healthier diet and undergo more closely medical follow-ups, including disease screening. Our high screening rate thus translates into a lower likelihood of bias by PCa detection than many other studies. Moreover, we had the ability to conduct a sensitivity analysis excluding controls who were not recently screened to reduce the potential of latent cases in our controls series. This had a minimal impact on our findings given the high proportions of screened controls. In a cohort study conducted by Shin et al., associations with dietary patterns differed when screening or subjective symptoms were considered [23].

## 5. Conclusions

We observed that men adhering to a Healthy Eating pattern had a lower risk of overall and high-grade PCa, while the Western Sweet and Beverages pattern was associated with increased risks. These associations followed exposure-response patterns. Since as yet no modifiable risk factor has been clearly identified for this cancer, our findings provide valuable evidence towards the establishment of preventive measures.

## Figures and Tables

**Table 1:** Selected characteristics of cases and controls participating in the PROtEuS, Montreal, Canada, 2005–2012.

Characteristics	Cases		Controls		*p*-Value
	(*n* = 1919)		(*n* = 1991)		
Age in years, mean (SD)	64	(6.8)	65	(6.9)	<0.001
Ancestry, n (%)					<0.001
Black	128	(6.7)	89	(4.5)	
Asian	24	(1.3)	72	(3.6)	
European	1693	(88.2)	1699	(85.3)	
Greater Middle Eastern	45	(2.3)	100	(5.0)	
Latino	29	(1.5)	31	(1.6)	
Family income in $ CAD, n (%)					0.54
<20,000	223	(11.6)	245	(12.3)	
20,000–29,999	262	(13.7)	252	(12.7)	
30,000–49,999	445	(23.2)	462	(23.2)	
50,000–79,999	422	(22.0)	410	(20.6)	
>80,000	425	(22.1)	428	(21.5)	
Unknown	142	(7.4)	194	(9.7)	
Education, *n* (%)					0.34
Primary school of less	443	(23.1)	426	(21.4)	
High school	572	(29.8)	578	(29.0)	
College	313	(16.3)	375	(18.8)	
University	588	(30.6)	610	(30.6)	
Other	3	(0.2)	2	(0.1)	
Body mass index (kg/m^2^), mean (SD)	26.8	(4.0)	27.2	(4.4)	0.003
Ever smoked, *n* (%)					0.23
No	514	(26.8)	514	(25.8)	
Yes	1404	(73.2)	1477	(74.2)	
Overall physical activity, *n* (%)					0.55
Not very active	432	(22.5)	488	(24.5)	
Moderately active	522	(27.2)	558	(28.0)	
Very active	965	(50.3)	945	(47.5)	
Last prostate screening test, *n* (%)					< 0.001
≤2 years before index date	1903	(99.2)	1510	(75.8)	
>2 years before index date	1	(0.02)	235	(11.8)	
Never screened	2	(0.1)	190	(9.5)	
Unknown	13	(0.7)	56	(2.8)	
First-degree relative with prostate cancer, *n* (%)					< 0.001
No	1409	(73.4)	1736	(87.2)	
Yes	447	(23.3)	199	(10.0)	
Unknown	63	(3.3)	56	(2.8)	
Use of vitamins or mineral supplements, *n* (%)					0.08
No	1184	(61.7)	1222	(61.4)	
Yes	735	(38.3)	768	(38.6)	
Total calories 2 years ago (kcal/day), mean (SD)	1989.0	(663.4)	1916.9	(645.6)	0.08
Proxy respondent, *n* (%)	49.0	(2.6)	76.0	(3.8)	0.16

**Table 2:** Weekly intake of 68 food and beverage items and rotated factor loadings for food and beverage items loadings having absolute values of ≥0.2 for any factor.

	Servings per			Rotated Factor Loadings		
Food and Beverage Items	Week ^1^			Pattern 1	Pattern 2	Pattern 3
Banana	2.69	±	2.71	0.29		0.22
Apple, pear	2.84	±	3.38	0.55		
Orange, grapefruit, other citrus fruits	2.32	±	2.94	0.47		
Peaches, nectarine	0.61	±	1.31	0.54		
Canned fruit, fruit sauce, fruit salad	0.62	±	1.50			0.45
Apricots	0.26	±	0.95	0.44		
Cantaloupe	0.48	±	0.89	0.52		
Watermelon, honeydew melon	0.38	±	0.65	0.48		
Strawberries, raspberries, blueberries	1.10	±	1.52	0.45		0.28
Other fresh fruit	1.58	±	1.84	0.50		
Potatoes, fried or pan fried	0.72	±	0.98		0.43	0.28
Potatoes, not fried	2.51	±	2.24			0.33
Sweet potatoes	0.17	±	0.59	0.29		
Baked beans, other legumes or lentils	0.86	±	1.16	0.37		
Broccoli	1.27	±	1.34	0.50		
Carrots	2.01	±	1.87	0.33		0.28
Spinach	0.51	±	0.81	0.56		
Coleslaw, cabbage, cauliflower, Brussel’s sprouts	0.93	±	1.12	0.35		
Dark lettuce	2.42	±	2.26	0.48		
Tomatoes	3.08	±	2.45	0.37		
Sweet red peppers	0.97	±	1.43	0.49		
Other vegetables	2.81	±	2.16	0.34		
Tomato soup or cream of tomato	0.34	±	0.68			0.40
Vegetable soup	1.25	±	1.50	0.22		0.30
Tofu, soybeans	0.17	±	0.66	0.26	−0.23	
Ketchup, salsa	0.83	±	1.35		0.22	0.43
Salad dressing, mayonnaise (excl. low fat)	2.24	±	2.36		0.27	0.27
Beef	1.89	±	1.42		0.49	
Pork	1.03	±	0.90		0.46	
Chicken, turkey, or other poultry	1.89	±	1.10		0.21	
Veal, lamb	0.40	±	0.61	0.39	0.26	
Liver	0.17	±	0.29			
Hot-dogs or sausage	0.42	±	0.66		0.50	0.27
BBQ	1.14	±	1.40	0.23	0.48	
Cold cuts	1.23	±	1.64		0.48	0.28
Bacon, breakfast sausage	0.49	±	0.90		0.46	0.31
Fish, seafood	1.29	±	1.02	0.43		
Eggs, omelets, or quiche	1.79	±	1.87			0.28
Cheese	3.80	±	2.76	0.22	0.26	
Pasta with tomato sauce	1.07	±	0.93		0.25	0.29
Pasta with cheese without tomato sauce	0.23	±	0.41			0.21
Pizza	0.40	±	0.56		0.35	0.23
Cookies, muffins	2.44	±	3.31			0.50
White bread	5.52	±	8.16	−0.30	0.35	
Brown bread	5.86	±	7.33	0.45	−0.24	
Rice	1.68	±	1.91	0.37		
Donuts, cakes, pastries, and pies	1.39	±	2.24			0.50
Oatmeal or cream of wheat	0.63	±	1.48		−0.23	0.32
Breakfast cereal	1.92	±	2.49	0.21	−0.23	0.43
Real fruit juice	3.76	±	4.05			0.28
Tomato or vegetable juice	1.07	±	1.84			0.39
Glass of milk or milk in cereal	4.64	±	5.74		−0.23	0.45
Cream or milk in coffee or tea	12.07	±	13.89		0.20	
Dark carbonated soft drinks	2.46	±	6.50	−0.27	0.26	0.31
Other carbonated soft drinks	0.85	±	2.66			0.21
Fried food	0.28	±	0.58		0.21	
Nuts or peanuts butter	2.74	±	2.86	0.26		0.23
Chips, corn chips, popcorn, tortillas	0.82	±	1.55		0.32	0.40
Chocolate	0.83	±	1.75			0.35
Yoghurt	2.33	±	2.97	0.42		0.20
Ice cream	0.82	±	1.38			0.40
Fat of beef or pork	1.44	±	31.66		0.28	
Meat slightly blackened	1.01	±	1.77		0.42	
Coffee	14.90	±	14.58		0.33	
Green tea	1.31	±	4.23	0.29		
Beer	3.52	±	9.21		0.38	
Wine	4.05	±	6.99	0.35	0.39	
Spirits	1.08	±	4.72	0.21	0.27	
Proportion of variance explained (%)				8.6	7.2	3.7
Cumulative variance explained (%)				8.6	15.8	19.5

^1^ No. of servings per week ± standard deviation.

**Table 3:** Odds ratios (OR) and 95% confidence intervals (CI) for the association between dietary pattern scores and prostate cancer risk, overall and by tumor grade, PROtEuS, Montreal, Canada, 2005–2012 ^1^.

Quartiles of Dietary Pattern Score	1991 Controls	All Prostate Cancers 1917 Cases	Low-Grade Prostate Cancers ^2^ 1385 Cases	High-Grade Prostate Cancers ^3^ 529 Cases
	*n*	*n* Cases/OR (95% CI)	*n* Cases/OR (95% CI)	*n* Cases/OR (95% CI)
Healthy Eating				
1	497	499/1.00 (reference)	335/1.00 (reference)	163/1.00 (reference)
2	499	505/0.95 (0.78–1.15)	378/1.07 (0.86–1.32)	126/0.75 (0.57–0.99)
3	497	477/0.84 (0.68–1.02)	343/0.91 (0.73–1.14)	133/0.77 (0.58–1.02)
4	498	436/0.76 (0.61–0.93)	329/0.90 (0.71–1.13)	107/0.66 (0.48–0.89)
		*p*_trend_ = 0.004	*p*_trend_ = 0.16	*p*_trend_ = 0.008
Western Salty and Alcohol				
1	498	457/1.00 (reference)	325/1.00 (reference)	132/1.00 (reference)
2	498	482/0.92 (0.76–1.13)	331/0.88 (0.70–1.10)	149/0.99 (0.75–1.32)
3	497	482/0.87 (0.71–1.06)	355/0.88 (0.71–1.10)	126/0.82 (0.61–1.10)
4	498	496/0.89 (0.72–1.09)	374/0.91 (0.73–1.14)	122/0.78 (0.58–1.06)
		*p*_trend_ = 0.21	*p*_trend_ = 0.59	*p*_trend_ = 0.05
Western Sweet and Beverages				
1	498	388/1.00 (reference)	285/1.00 (reference)	103/1.00 (reference)
2	498	442/1.01 (0.82–1.24)	325/0.96 (0.76–1.20)	117/0.96 (0.70–1.30)
3	498	494/1.12 (0.91–1.38)	360/1.01 (0.80–1.27)	134/1.06 (0.78–1.45)
4	497	593/1.35 (1.10–1.66)	415/1.14 (0.91–1.44)	175/1.32 (0.98–1.80)
		*p*_trend_ = 0.002	*p*_trend_ = 0.23	*p*_trend_ = 0.02

^1^ Adjusted for age, ancestry, education, first-degree family history of prostate cancer, and timing of last prostate cancer screening. ^2^ Prostate cancer cases with a Gleason score ≤ 6, or of 7 with a primary score of 3. ^3^ Prostate cancer cases with a Gleason score of 7 with a primary score of 4, or ≥ 8. Information on the primary or secondary score was missing for 3 cases.

## Supplementary Materials

The following are available online at https://www.mdpi.com/2072-6643/12/7/1907/s1, Table S1: Odds ratios (OR) and 95% confidence interval (CI) for the association between dietary patterns scores and prostate cancer risk, overall and by tumour grade, based on a minimal model for adjustment, PROtEuS, Montreal, Canada, 2005-20121., Table S2: Odds ratios (OR) and 95% confidence interval (CI) for the association between dietary patterns score and prostate cancer risk, overall and by tumour grade, excluding controls who had not been screened for PCa in the previous 2 years, PROtEuS, Montreal, Canada, 2005-20121.
