# Supplementary material for: Dietary Patterns Are Associated with Risk of Prostate Cancer in a Population-Based Case-Control Study in Montreal, Canada
Source: Nutrients. 2020 Jun 27;12(7):1907. doi: 10.3390/nu12071907 (PMC7399998; doi:10.3390/nu12071907)
Supplement: Supplementary file 1 [file nutrients-12-01907-s001.pdf]

Supplementary Files

Table S1. Odds ratios (OR) and 95% confidence interval (CI) for the association between dietary patterns scores and prostate cancer risk, overall and by tumour grade, based on a minimal model for adjustment, PROtEuS, Montreal, Canada, 2005-2012[1].

Quartiles of dietary pattern score	1 991 controls n	All prostate cancers 1917 cases n cases	OR (95% CI)	Low-grade prostate cancers[2] 1385 cases n cases	OR (95% CI)	High-grade prostate cancers[3] 529 cases n cases	OR (95% CI)
Healthy Eating							
1	497	499	1.00 (reference)	335	1.00 (reference)	163	1.00 (reference)
2	499	505	1.06 (0.88-1.29)	378	1.19 (0.96-1.46)	126	0.80 (0.61-1.10)
3	497	477	0.99 (0.81-1.21)	343	1.07 (0.86-1.34)	133	0.82 (0.62-1.01)
4	498	436	1.01 (0.82-1.24)	329	1.10 (0.89-1.39)	107	0.86 (0.64-1.15)
			P_{trend} = 0.87		P_{trend} = 0.71		P_{trend} = 0.27
Western Salty & Alcohol							
1	498	457	1.00 (reference)	325	1.00 (reference)	132	1.00 (reference)
2	498	482	0.87 (0.72-1.06)	331	0.81 (0.65-1.00)	149	1.04 (0.78-1.39)
3	497	482	1.00 (0.82-1.22)	355	1.00 (0.80-1.24)	126	0.98 (0.73-1.33)
4	498	496	0.84 (0.69-1.03)	374	0.82 (0.65-1.02)	122	0.89 (0.66-1.21)
			P_{trend} = 0.23		P_{trend} = 0.35		P_{trend} = 0.24
Western Sweet & Beverages							
1	498	388	1.00 (reference)	285	1.00 (reference)	103	1.00 (reference)
2	498	442	1.14 (0.93-1.38)	325	1.13 (0.92-1.41)	117	1.14 (0.84-1.53)
3	498	494	1.16 (0.96-1.41)	360	1.17 (0.94-1.45)	134	1.15 (0.85-1.56)
4	497	593	1.32 (1.09-1.61)	415	1.24 (1.00-1.55)	175	1.50 (1.12-2.03)
			P_{trend} = 0.007		P_{trend} = 0.05		P_{trend} = 0.006

[1]Adjusted for age, ancestry, education, and marital status.
[2]Prostate cancer cases with a Gleason score ≤ 6, or 7 with a primary score of 3.
[3]Prostate cancer cases with a Gleason score of 7 with a primary score of 4, or ≥ 8.

Table S2. Odds ratios (OR) and 95% confidence interval (CI) for the association between dietary patterns score and prostate cancer risk, overall and by tumour grade, excluding controls who had not been screened for PCa in the previous 2 years, PROtEuS, Montreal, Canada, 2005-2012[1].

Quartiles of dietary pattern score	1 242 controls	All prostate cancers 1816 cases		Low-grade prostate cancers[2] 1311 cases		High-grade prostate cancers[3] 502 cases	
	n	n cases	OR (95% CI)	n cases	OR (95% CI)	n cases	OR (95% CI)
Healthy Eating							
1	270	436	1.00 (reference)	287	1.00 (reference)	148	1.00 (reference)
2	311	481	0.95 (0.76-1.17)	363	1.08 (0.86-1.36)	117	0.70 (0.52-0.94)
3	322	440	0.83 (0.67-1.04)	323	0.91 (0.71-1.15)	116	0.67 (0.50-0.91)
4	339	459	0.84 (0.67-1.04)	338	0.91 (0.71-1.17)	121	0.69 (0.51-0.94)
			P_{trend} = 0.06		P_{trend} = 0.18		P_{trend} = 0.01
Western Salty & Alcohol							
1	306	462	1.00 (reference)	336	1.00 (reference)	126	1.00 (reference)
2	333	429	0.80 (0.65-0.99)	292	0.74 (0.59-0.94)	135	0.94 (0.70-1.27)
3	286	469	0.98 (0.79-1.22)	349	0.99 (0.78-1.25)	119	0.95 (0.69-1.29)
4	317	456	0.82 (0.65-1.02)	334	0.80 (0.63-1.01)	122	0.84 (0.61-1.15)
			P_{trend} = 0.26		P_{trend} = 0.43		P_{trend} = 0.24
Western Sweet & Beverages							
1	308	415	1.00 (reference)	303	1.00 (reference)	112	1.00 (reference)
2	321	443	1.03 (0.84-1.28)	326	1.05 (0.83-1.32)	117	1.03 (0.76-1.41)
3	307	469	1.17 (0.90-1.39)	348	1.14 (0.90-1.45)	121	1.09 (0.79-1.50)
4	306	489	1.21 (0.98-1.51)	334	1.16 (0.91-1.47)	152	1.39 (1.02-1.91)
			P_{trend} = 0.06		P_{trend} = 0.20		P_{trend} = 0.01

[1] Adjusted for age, ancestry, education, first-degree family history of prostate cancer, and timing of last prostate cancer screening.
[2]Prostate cancer cases with a Gleason score ≤ 6, or 7 with a primary score of 3.
[3]Prostate cancer cases with a Gleason score of 7 with a primary score of 4, or ≥ 8.
